# MiR-424/503-Mediated Rictor Upregulation Promotes Tumor Progression

**DOI:** 10.1371/journal.pone.0080300

**Published:** 2013-11-11

**Authors:** Chitose Oneyama, Yoriko Kito, Rei Asai, Jun-ichiro Ikeda, Takuya Yoshida, Daisuke Okuzaki, Rie Kokuda, Kyoko Kakumoto, Ken-ichi Takayama, Satoshi Inoue, Eiichi Morii, Masato Okada

**Affiliations:** 1 Department of Oncogene Research, Research Institute for Microbial Diseases, Osaka University, Suita, Osaka, Japan; 2 Department of Pathology, Graduate School of Medicine, Osaka University, Suita, Osaka, Japan; 3 Laboratory of Biophysical Chemistry, Graduate School of Pharmaceutical Sciences, Osaka University, Suita, Osaka, Japan; 4 DNA-chip Developmental Center for Infectious Diseases, Research Institute for Microbial Diseases, Osaka University, Suita, Osaka, Japan; 5 Department of Anti-Aging Medicine, Graduate School of Medicine, The University of Tokyo, Bunkyo-ku, Tokyo, Japan; 6 Department of Geriatric Medicine, Graduate School of Medicine, The University of Tokyo, Bunkyo-ku, Tokyo, Japan; 7 Division of Gene Regulation and Signal Transduction, Research Center for Genomic Medicine, Saitama Medical University, Hidaka, Saitama, Japan; University of Kentucky College of Medicine, United States of America

## Abstract

mTOR complex 2 (mTORC2) signaling is upregulated in multiple types of human cancer, but the molecular mechanisms underlying its activation and regulation remain elusive. Here, we show that microRNA-mediated upregulation of Rictor, an mTORC2-specific component, contributes to tumor progression. Rictor is upregulated via the repression of the miR-424/503 cluster in human prostate and colon cancer cell lines that harbor c-Src upregulation and in Src-transformed cells. The tumorigenicity and invasive activity of these cells were suppressed by re-expression of miR-424/503. Rictor upregulation promotes formation of mTORC2 and induces activation of mTORC2, resulting in promotion of tumor growth and invasion. Furthermore, downregulation of miR-424/503 is associated with Rictor upregulation in colon cancer tissues. These findings suggest that the miR-424/503–Rictor pathway plays a crucial role in tumor progression.

## Introduction

The evolutionarily conserved Ser/Thr kinase mTOR (mammalian target of rapamycin) plays pivotal roles in regulating cell growth, proliferation, and survival [1,2]. Dysregulation of mTOR signaling is frequently observed in many types of cancers, implicating it in promotion of tumor growth and malignancy [3-5]. mTOR assembles with alternative binding partners to generate two functionally distinct protein complexes: mTOR complex 1 (mTORC1), containing Raptor, and mTOR complex 2 (mTORC2), containing Rictor [6,7]. mTORC1 controls cell growth by regulating mRNA translation via phosphorylation of its downstream substrates, ribosomal S6 kinase (S6K) and 4E binding protein 1 (4E-BP1) [8,9]. By contrast, mTORC2 regulates cell proliferation, survival and actin cytoskeleton by activating AKT, protein kinase C-α (PKC-α) and serum-glucocorticoid-induced protein kinase-1 (SGK1) [7,10-12]. Although both complexes are activated by growth factor signaling, the signaling cascade leading to activation and regulation of mTORC2 are considerably less known compared to those of mTORC1.

The mTORC2 complex consists of mTOR, Rictor, mLST8, mSin1, Protor, and Deptor [13,14]. Overexpression of Rictor, a specific component of mTORC2, is observed in some cancers such as gliomas, and its forced expression promotes mTORC2 assembly and activity, conferring increased proliferative and invasive potential on cancer cells [15]. In mice that lack the tumor suppressor PTEN, mTORC2, and more particularly Rictor, is required for the development of prostate cancer [16]. In melanoma and colon cancer cells, mTORC2-ribosome association is important in oncogenic PI3K signaling [17]. Although these recent studies indicate that mTORC2 plays important roles in cancer signaling, little is known about the signaling cascade leading to mTORC2 activation and regulation. 

Recently, potential roles have been proposed for microRNAs (miRNAs) in the regulation of mTORC2 components. miRNAs are non-coding small RNA molecules that control diverse cellular functions, such as cell proliferation and differentiation, by regulating expression of target genes. Because dysregulation of miRNA expression is associated with a variety of human cancers, specific miRNAs can be considered to act as oncogenes or tumor suppressors [18,19]. For example, miR-100 and miR-199a-3p suppress mTOR expression [20-22], and miR-152 and miR-218 suppress Rictor in some cancers [23,24]. These findings suggest that mTORC2 function can be regulated by a set of miRNAs under the control of oncogenic signals.

The tyrosine kinase c-Src is upregulated in various human cancers and plays a crucial role in tumor progression [25-29]. Once activated by extracellular signals such as EGF, c-Src acts as a common upstream regulator of multiple oncogenic pathways, including the Ras/MAPK and PI3K/AKT pathways, thereby inducing tumor progression [30]. In normal cells, the kinase activity of c-Src, corresponding to the phosphorylation at Y418 in human sequence, is rigorously controlled by the C-terminal Src kinase (Csk) [31]; therefore, the oncogenic potential of c-Src is suppressed. We have investigated the mechanism of c-Src–mediated tumorigenesis using Csk-deficient fibroblasts (*Csk* ^*-/-*^ cells), which can be transformed by wild-type c-Src, as a model system [27]. In previous work using this system, we investigated c-Src–induced tumor progression, focusing on the roles of microRNAs. Recently, we showed that miR-99a, which is downregulated by the activation of Src-related oncogenic pathways, controls mTOR expression in various human cancers. This novel regulatory role of miR-99a suggests a missing link between Src and mTOR in cancer progression [32]. Earlier studies suggested that Src-induced cell transformation is mediated via the mTOR signaling pathway [33,34], but the mechanism underlying Src-mediated activation of mTOR signaling remains to be addressed.

To elucidate the molecular link between the activation of the Src-related oncogenic pathway and mTOR-mediated tumor progression, we investigated the expression of mTOR complex components and the activity of downstream signaling molecules in human colon and prostate cancer cells in which the Src pathway is activated. In cancer cells and Src-transformed fibroblasts, we found that Rictor is upregulated via the repression of the miR-424/503 cluster, resulting in promotion of mTORC2 formation and activation implicated in cell proliferation and migration. Furthermore, the significant correlation of downregulation of miR-424/503 and Rictor upregulation in human colon cancer cells strongly suggests that the upregulation of the miR-424/503–Rictor pathway is crucial for promoting growth and invasive potential of various human cancers. 

## Results

### Rictor upregulation is associated with tumor growth

**Figure 1:**
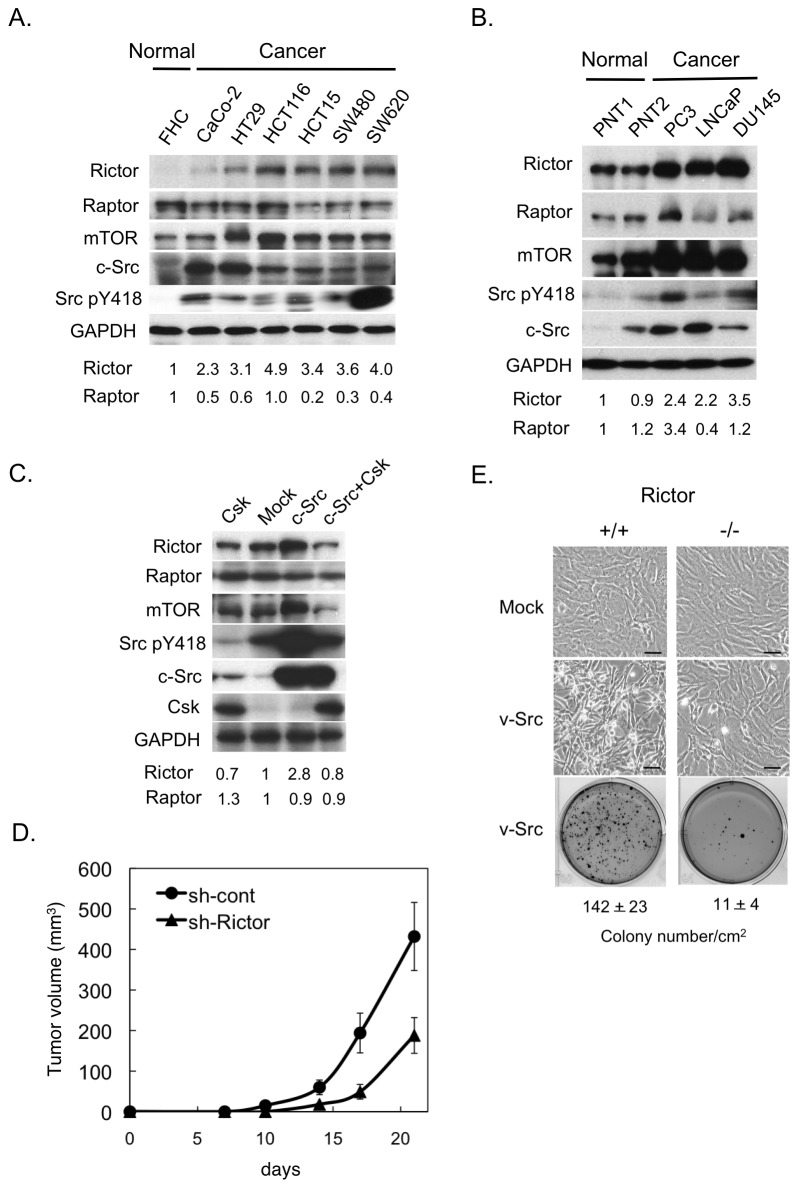
Rictor upregulation in human cancer cells and c-Src–transformed cells. (**A** and **B**) Whole-cell lysates from the indicated colon (A) and prostate (B) cancer cells were immunoblotted with the indicated antibodies. Data were obtained from three independent experiments and analyzed by one-way analysis of variance (ANOVA), followed by Bonferroni post-hoc tests using GraphPad Prism (GraphPad Software Inc., San Diego, CA, USA). Rictor upregulation in cancer cells were validated (P<0.05, FHC versus colon cancer cells; P<0.05, PNT1 or PNT2 versus prostate cancer cells). The difference of Rictor levels between normal prostate cells (PNT1 and PNT2) was not significant (P>0.05). (**C**) Whole-cell lysates from *Csk* ^*-/-*^ cells expressing Csk [Csk], empty vector [mock], c-Src [c-Src], and c-Src plus Csk [c-Src+Csk]) were subjected to immunoblotting with the indicated antibodies. (**D**) *Csk* ^*-/-*^/c-Src cells expressing control (sh-cont) or Rictor shRNA #1 (sh-Rictor) were inoculated subcutaneously into nude mice. Averages ± S.D. of tumor volume (mm^3^) obtained from four mice are plotted versus days after inoculation. (**E**) MEF (Rictor^+/+^) and Rictor-deficient MEFs (Rictor^-/-^) were infected with retrovirus expressing v-Src, and cell morphology at a magnification of 200× and soft-agar colony-formation activity were analyzed. *Scale* *bar* = 20 μm. The mean numbers of colonies per cm^2^ ± S.D. obtained from three independent experiments are shown. Relative levels of expression of Rictor and Raptor are shown below panels A-C.

We first investigated the expression of distinctive mTOR complex components in several lines of human colon and prostate cancer cells in which Src is upregulated. Western-blot analysis of whole-cell lysates revealed significant (P<0.05) upregulation of Rictor in all colon and prostate cancer cell lines tested (Figure 1A and 1B). By contrast, there was no significant change in the expression of Raptor. In these cells, we also observed upregulation of mTOR protein, consistent with our previous observations [32]. We next examined the effects of Src transformation on Rictor expression. For this experiment, we used Csk-deficient fibroblasts (*Csk* ^*-/-*^ cells), which can be transformed by wild-type c-Src [27]. Using these cells, we found that Rictor and mTOR, but not Raptor, were dramatically upregulated by c-Src–induced cell transformation (Figure 1C). Similarly, c-Src activation by EGF stimulation induced upregulation of Rictor and mTOR (Figure S1A). These findings suggest that Rictor is preferentially upregulated in various human cancer cell lines, as well as in Src-transformed cells.

To assess the role of Rictor upregulation, we examined the effects of short hairpin RNA (shRNA) knockdown of Rictor on Src transformation. Rictor knockdown efficiently suppressed anchorage-independent growth in c-Src transformed cells (Figures S1B and S1C). Tumorigenesis of c-Src–transformed cells in nude mice was also suppressed by Rictor knockdown (Figure 1D). Furthermore, Rictor^-/-^ MEFs were relatively resistant to v-Src–induced transformation, as compared to Rictor^+/+^ cells (Figures 1E and S1D). These results indicate that Rictor upregulation is tightly associated with promotion of cell transformation as well as tumor growth. 

The effect of Rictor upregulation on the formation of the mTOR complex was confirmed by immunoprecipitation assays. mTOR was co-precipitated with Rictor in quantities that paralleled the levels of Rictor expression. By contrast, Raptor was not co-precipitated with Rictor, and the interaction of mTOR with Raptor (i.e., mTORC1 formation) was not affected by Rictor expression (Figure S1E). These observations suggest that upregulated Rictor contributes to promotion of tumor growth by enhancing mTORC2 formation. 

### Rictor expression is regulated by the miR-424/503 cluster

To address the mechanism for Rictor upregulation, we focused on our previous observations in c-Src–transformed cells, where c-Src upregulation induced selective downregulation of a set of potentially tumor-suppressive miRNAs [32]. The bioinformatic search (TargetScan) analysis predicted that Rictor is included in the conserved targets of one of the downregulated miRNAs, the miR-424/503 cluster. We thus investigated the role of the miR-424/503 cluster in regulation of Rictor expression.

**Figure 2:**
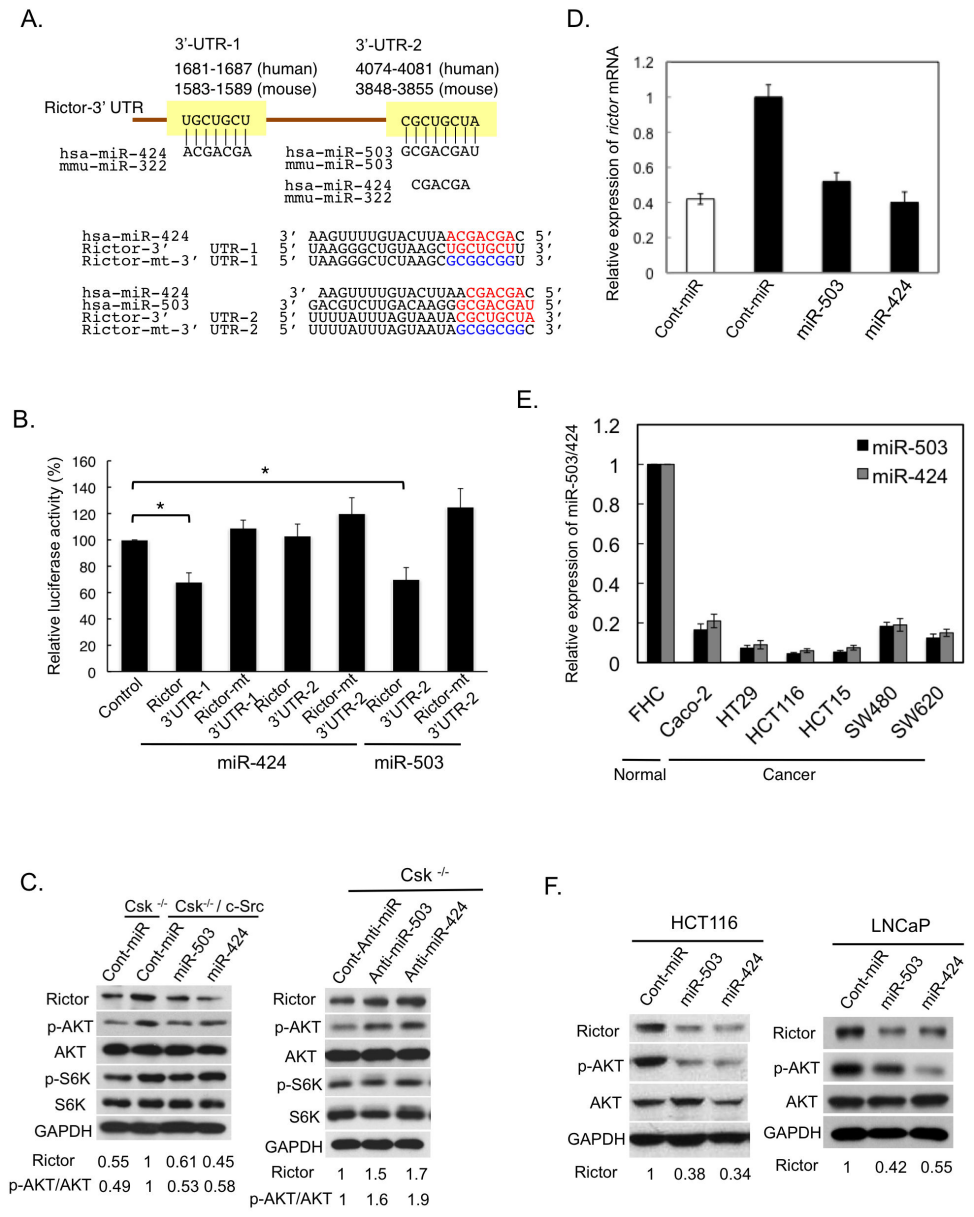
Rictor expression is regulated by the miR-424/503 cluster. (**A**) Alignment of RNA sequences of hsa-miR-424/503 (corresponding to mmu-miR-322/503 in mouse), and potential miR-424/503–binding sequences in Rictor 3’-UTRs. The seed sequence of miR-424/503 and its binding sequences are shown in red; mutated sequences are shown in blue. (**B**) pMIR-Rictor-1 and pMIR-Rictor-2 luciferase reporter constructs, containing either wild-type or mutated (mt) Rictor 3’UTR-1 and 3’UTR-2, were co-transfected with 30 nM of control, miR-503, or miR-424 into c-Src–transformed cells. Relative Renilla luciferase expression was standardized to a transfection control. The mean values of relative luciferase activity ± S.D. were obtained from three independent assays. *, p < 0.05 by Student’s t-test. (**C**) *Csk* ^*-/-*^ cells and c-Src–transformed cells (*Csk* ^*-/-*^/c-Src) were transfected with 30 nM of control, miR-503, or miR-424 (left panels). *Csk* ^*-/-*^ cells were transfected with 30 nM of control, anti-miR-503, or anti-miR-424 (right panels). Whole-cell lysates were immunoblotted with the indicated antibodies. (**D**) Csk^-/-^ and Csk^-/-^/c-Src cells were transfected with 30 nM of control, miR-503, or miR-424. The expression of *RICTOR* mRNA was analyzed by real-time PCR. (**E**) The expression levels of miR-503 (black) and miR-424 (grey) in the indicated colon cell lines were assessed by qRT-PCR. (**F**) Whole-cell lysates from HCT116 and LNCaP cells transfected with 30 nM of control, miR-503, or miR-424 were immunoblotted with the indicated antibodies. The relative expression levels of Rictor are shown at the bottom of the panels (C, D, and F).

 As shown in Figure 2A, the 3’-untranslated region (3’-UTR) of human *RICTOR* mRNA contains two potential miR-424 binding sequences (3’-UTR-1; 1681–1687 and 3’-UTR-2; 4074–4081), the latter of which (3’-UTR-2) partly overlaps with a potential miR-503 binding sequence. However, a luciferase reporter assay in c-Src–transformed cells revealed that miR-424 and miR-503 selectively target 3’-UTR-1 and 3’-UTR-2, respectively (Figure 2B). By the expression of miR-503 or miR-424, Rictor protein was downregulated as well as the activity of AKT, a critical downstream effector of mTORC2, whereas phosphorylation of the mTORC1 target S6K was not affected in c-Src–transformed cells (Figure 2C, left panels); this indicates that miR-424/503-mediated Rictor specifically controls the mTORC2 pathway. Inversely, functional knockdown using anti-miR-503 or anti-miR-424 increased Rictor expression and the activity of AKT (Figure 2C, right panels). qRT-PCR analyses revealed that transfection with miR-503 or miR-424 reduced the level of *RICTOR* mRNA in parallel with Rictor protein level, suggesting that Rictor expression is downregulated by miRNA-mediated mRNA degradation (Figure 2C and 2D). These findings demonstrate that Rictor is directly targeted by miR-424/503 and suggest that downregulation of miR-424/503 by c-Src activation is tightly associated with the upregulation of mTORC2 in c-Src-transformed cells.

To determine whether Rictor is regulated by miR-424/503 in human cancers, we measured the expression of miR-424/503 in several human colon cancer cell lines that overexpress Rictor (Figure 1A). qRT-PCR analyses revealed that the expression of miR-424/503 was greatly reduced in most colon cancer cell lines relative to the level in normal cells (Figure 2E), suggesting an inverse correlation between Rictor and miR-424/503 expressions. The ectopic expression of miR-424/503 in HCT116 colon cancer cells and LNCaP prostate cancer cells was able to suppress the expression of Rictor and the activity of mTORC2 in these cells (Figure 2F). These observations suggest that miR-424/503 can target Rictor and controls mTORC2 activity even in human cancers.

### The miR-424/503 cluster regulates tumor growth

**Figure 3:**
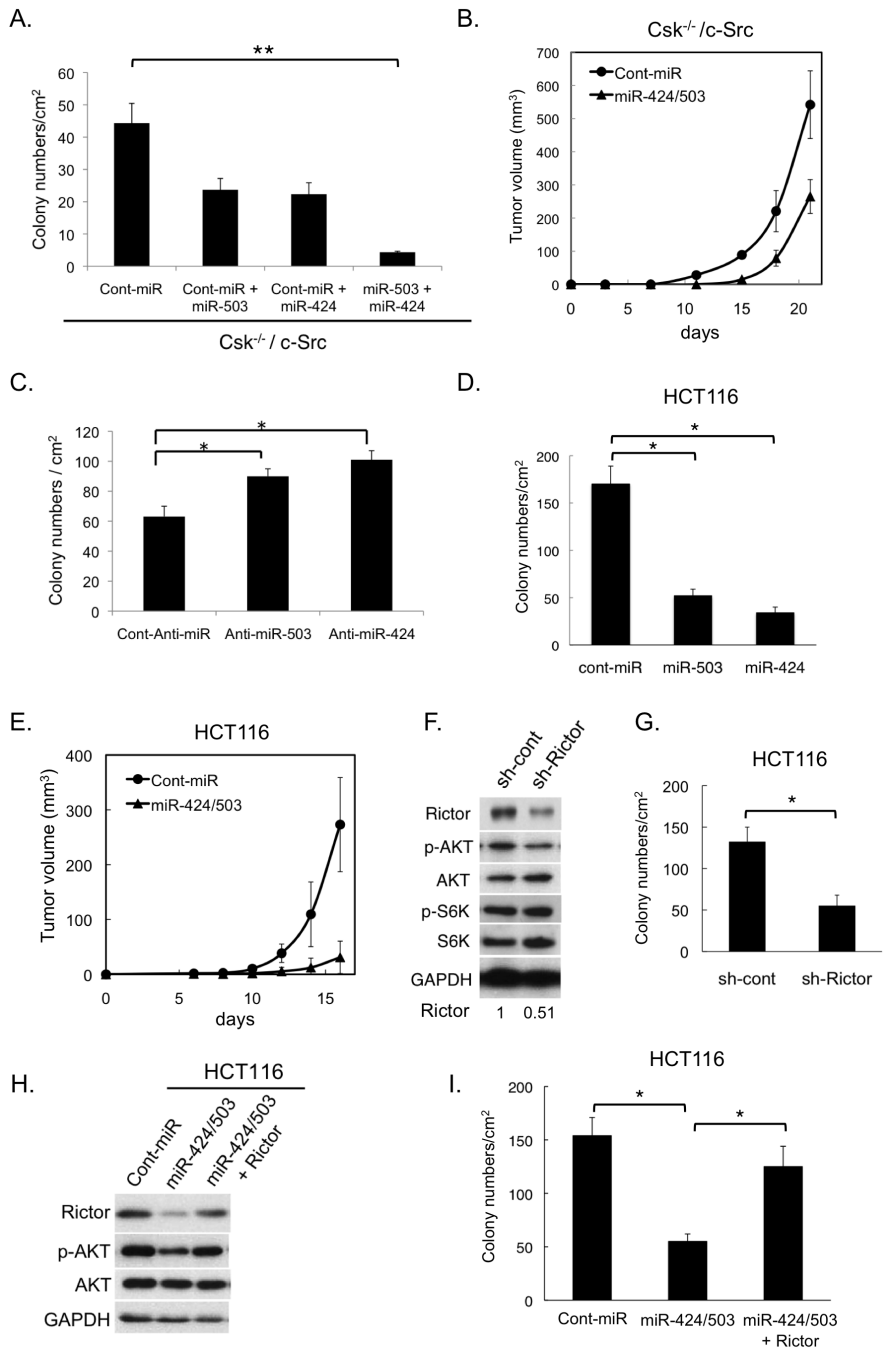
miR-424/503 cluster as a suppressor of tumor growth. (**A**) Csk^-/-^/c-Src cells were transfected with 5 nM of control, miR-503 and/or miR-424 and subjected to the soft-agar colony-formation assay for 7 days. (**B**) Csk^-/-^/c-Src cells treated with 15 nM each of miR-503 and miR-424, or 30 nM control, were inoculated subcutaneously into nude mice. Averages ± S.D. of tumor volume (mm^3^) obtained from five mice are plotted versus days after inoculation. (**C**) Csk^-/-^ cells were transfected with the 30 nM of control, anti-miR-503, or anti-miR-424 and subjected to the soft-agar colony-formation assay for 21 days. (**D**) HCT116 cells were treated with 30 nM of miR-503, miR-424, or cont-miR and subjected to the soft-agar colony-formation assay for 8 days. (**E**) HCT116 cells treated with 15 nM each of miR-503 and miR-424, or 30 nM control, were inoculated subcutaneously into nude mice. Averages ± S.D. of tumor volume (mm^3^) obtained from four mice are plotted versus days after inoculation. (**F**) HCT116 cells were expressed with control (sh-cont) or Rictor shRNA (sh-Rictor). Whole-cell lysates were immunoblotted with the indicated antibodies. Relative levels of Rictor expression are shown below panels. (**G**) Colony-forming activity of HCT116 cells expressing control (sh-cont) or Rictor shRNA. (**H**) HCT116 cells were treated with 15 nM each of miR-503 and miR-424, or 30 nM control with or without Rictor transfection, and the total cell lysates were immunoblotted with the indicated antibodies. (**I**) HCT116 cells indicated in (H) were subjected to soft-agar colony-formation assay. Colonies were scored 8 days after plating. The mean number of colonies ± S.D. was obtained from three independent experiments (A, C, D, G and I). *, p < 0.05 and **, p < 0.01 by Student’s t test.

We next investigated the contribution of miR-424/503 to tumor growth. Overexpression of miR-503 and 424 significantly suppressed anchorage-independent growth of c-Src–transformed cells in an additive manner, because the two miRNAs independently recognize different sites in the 3’-UTR of *RICTOR* mRNA (Figure 3A). Tumorigenesis of c-Src–transformed cells in nude mice was also significantly suppressed by the expression of miR-424/503 (Figure 3B). Inversely, when miR-424/503 was inactivated using anti-miR-424/503, untransformed *Csk* ^*-/-*^ cells acquired the ability to form colonies in soft agar (Figure 3C). Furthermore, the expression of miR-424/503 in HCT116 cells significantly suppressed not only *in vitro* tumor growth (Figure 3D) but also *in vivo* tumorigenesis (Figure 3E). The importance of Rictor upregulation in tumor growth was further confirmed by observing that shRNA-mediated Rictor knockdown in HCT116 cells significantly suppressed colony-forming activity (Figure 3F and 3G). Similar effects of miR-424/503 and Rictor knockdown on colony-forming activity were also observed in LNCaP cells (Figures S2A and S2B). However, when Rictor open reading frame (ORF) cDNA was introduced into miR-424/503-treated HCT116 cells, the colony-forming activity was considerably rescued (Figure 3H and 3I). These results suggest that the miR-424/503 cluster can function as a suppressor of tumor growth via repressing Rictor expression.

### The miR-424/503 regulates invasive potential

**Figure 4:**
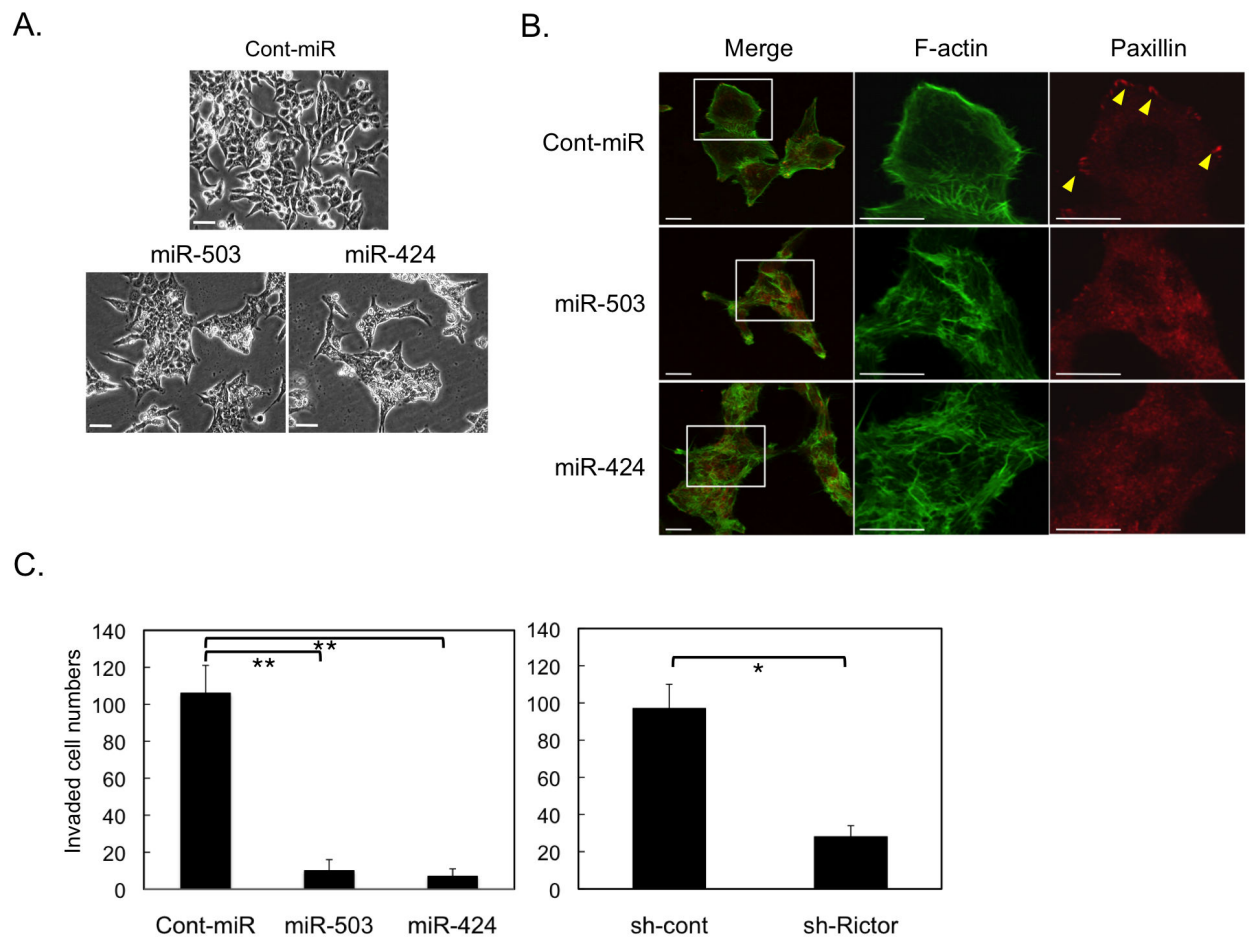
Role of the miR-424/503–Rictor pathway in cytoskeletal organization and invasive activity of human colon cancer cells. (**A**) The morphology of HCT116 cells expressing 30 nM of miR-424, -503, or control was observed by phase-contrast microscopy at a magnification of 200×. *Scale* *bar* = 50 μm. (**B**) Cells indicated in (A) were subjected to immunocytochemistry. F-Actin (green) and Paxillin (red) were analyzed by immunostaining of the indicated cells grown on fibronectin-coated dishes. Boxed images are enlarged in right panels. Locations of focal contacts are indicated by arrowheads. *Scale* *bar* = 20 μm. (**C**) *In* *vitro* invasiveness of HCT116 cells transfected with the indicated materials was analyzed. Cells (1.0 × 10^5^) were seeded into Matrigel invasion chambers. After 48 h, membranes were detached; cells were stained and counted. The mean number of cells per mm^2^ ± S.D. was obtained from three independent experiments. *, p < 0.05 and **, p < 0.01 by Student’s t-test.

In addition to the effects on tumor growth, we also recognized that expression of miR-424/503 induced a dramatic change in cell morphology in HCT116 cells (Figure 4A). Staining for F-actin and paxillin, a marker of focal contact, revealed that miR-424/503 transfection induced disruption of polarized stress fibers and a reduction in the numbers of focal contacts (Figure 4B). These findings suggest that the miR-424/503 can also affect cytoskeletal organization. 

 The dramatic effects of miR-424/503 treatment on cytoskeletal organization and formation of focal adhesions suggest that the miR-424/503 might also regulate the invasive potential of cancer cells. We therefore assessed the effects of perturbation of the pathway on the *in vitro* invasion potential of HCT116 cells in Matrigel-based assays. The introduction of miR-424/503 potently suppressed the invasive activity of HCT116 cells. Similar effects were also observed by shRNA-mediated knockdown of Rictor in these cells. (Figure 4C). These findings suggest that the miR-424/503-mediated Rictor function plays crucial roles in controlling cytoskeletal organization, formation of focal contacts and invasive potential of cancer cells.

### The miR-424/503–Rictor pathway in human cancers

**Figure 5:**
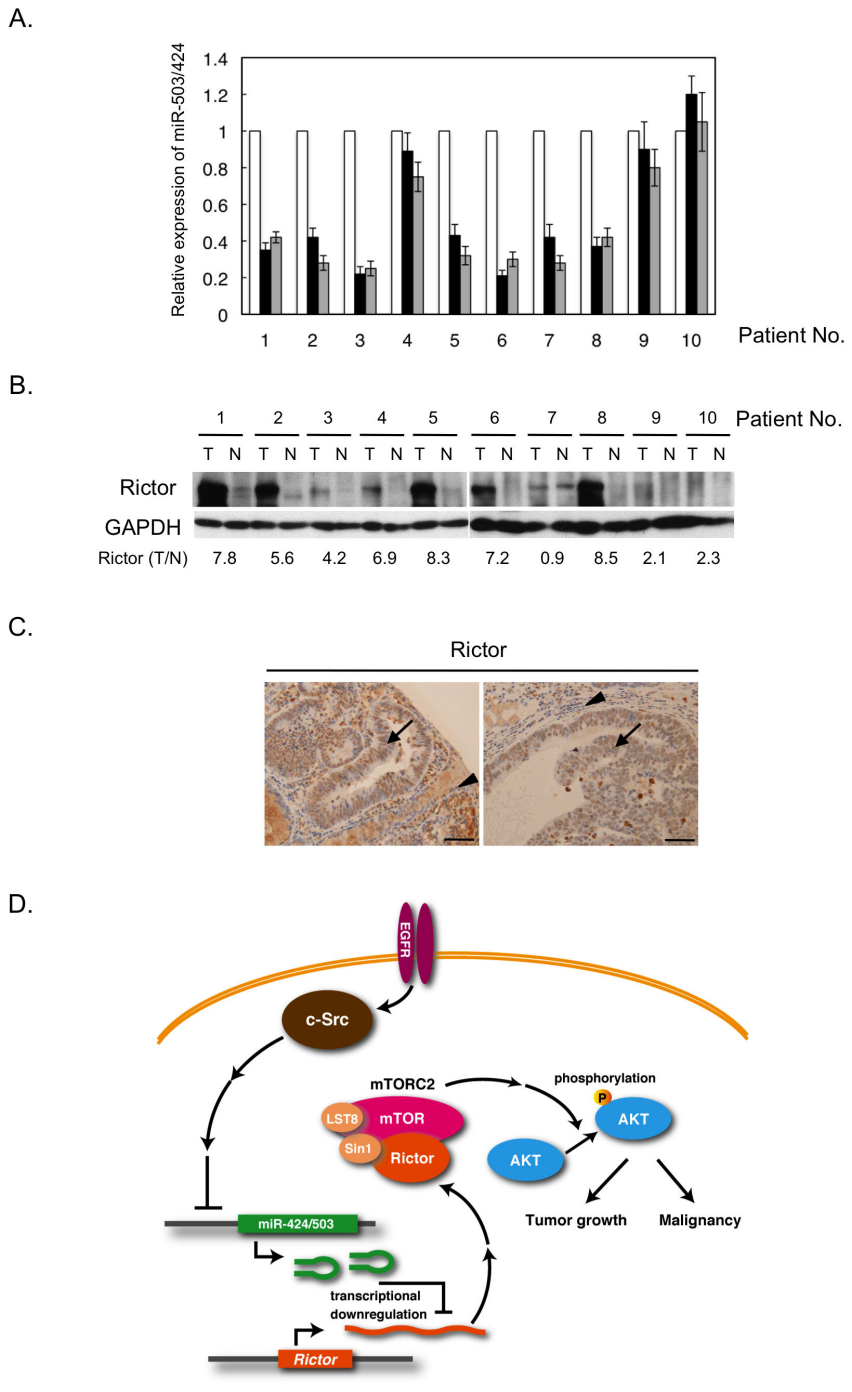
miR-424/503–Rictor pathway in human colon cancer tissues. (**A**) The relative expression levels of miR-503 (black) and miR-424 (grey) in colon tumors in comparison with adjacent non-cancerous tissues (white) were assessed by qRT-PCR. The mean values ± S.D. were obtained from three independent experiments. (**B**) Whole-tissue lysates from colon tumors (T) and adjacent non-cancerous tissues (N) indicated in (A) were analyzed by immunoblotting with the indicated lysates. (**C**) Immunohistochemistry for Rictor (200×) in colon adenocarcinoma (arrow) and non-cancerous (arrowhead) tissue samples. Representative images from 19 of 20 samples are shown. Scale bar corresponds to 100 μm. (**D**) A schematic model of the role of c-Src–miR-424/503–Rictor pathway. The downregulation of miR-424/503 mediated by Src activation results in the upregulation of Rictor, which in turn induces mTORC2 formation, leading to the promotion of tumor growth and progression.

Finally, we verified the role of the miR-424/503–Rictor pathway in human cancers by determining the expression levels of the pathway’s components in primary colon tumors as well as adjacent non-cancerous tissues. qRT-PCR analyses revealed that the expression of the miR-424/503 cluster was significantly downregulated in seven of the ten tumor samples we tested (Figure 5A), indicating that downregulation of miR-424/503 is associated with some types of human colon cancers. Western-blot analysis of these samples supported that there is a significant correlation between miR-424/503 repression and Rictor expression even in human cancer tissues (Figure 5B). To examine whether Rictor upregulation is observed in human cancer tissues, immunohistochemistry for Rictor was performed in 20 other colon tumor specimens. Compared to adjacent non-cancerous tissues, levels of Rictor were upregulated in 19 of 20 primary tumor regions (Figure 5C). These observations suggest that downregulation of miR-424/503 promotes cancer progression via Rictor upregulation in various human cancers. 

## Discussion

In this study, we identified the miR-424/503–Rictor pathway as a crucial pathway involved in the tumor progression. A schematic of the function of this axis is depicted in Figure 5D. In this model, when oncogenic signaling is initiated by upregulation of oncogenic molecules, e.g., c-Src, expression of the miR-424/503 cluster is specifically repressed. Rictor, a target of miR-424/503, is consequently upregulated, promoting mTORC2 formation. Then, activated mTORC2 induces activation of AKT to promote tumor growth as well as invasive activity of cancer cells. 

miR-424 and miR-503 are co-transcribed as a polycistronic primary transcript (pri-miRNA) and thus comprise the miR-424/503 gene cluster [35]. The mature miRNAs recognize different regions in 3'UTR of *RICTOR* mRNA and act additively (Figure 3A). Although the mechanism for transcriptional regulation of this gene cluster remains unclear, formation of miRNA clusters whose members share common target genes might still be evolutionarily advantageous, as small changes in cluster expression would exert an amplified effect on the mRNA targets of individual miRNAs. miR-424/503 has been reported to target several genes such as cdc25A [36], FGF2 and FGFR1 [37]. While we cannot exclude the possibility that miR-424/503 act via these targets, we found that the growth-suppressive effect of miR-424/503 is largely attributed to Rictor downregulation in colon cancer cells. Two other miRNAs that also target Rictor, miR-218 and miR-152, has shown to be downregulated in oral squamous cell carcinoma and endometrial cancer [23,24]. Taken together, it is possible that miRNA-mediated Rictor upregulation contributes to tumor progression in a wide array of human cancers. 

Previously, we identified several tumor-suppressive miRNAs that are repressed in c-Src–transformed cells: miR-99a targets mTOR to suppress tumor progression, and miR-542-3p targets ILK to suppress tumor malignancy [32,38]. miR-99a–mediated mTOR upregulation collaborates with miR-424/503–mediated Rictor upregulation to promote mTORC2 activation. Furthermore, it is possible that upregulation of ILK by repression of miR-542-3p also contributes to the activation of invasive potential induced via the miR-424/503–Rictor pathway. The direct interaction between ILK and Rictor further supports a functional interaction between these molecules [39]. Based on these findings, we suspect that mTOR complex components and their interacting molecules are convergent targets of tumor-suppressive microRNAs controlled by oncogenes, such as Src and EGF. This in turn highlights crucial roles of the mTOR pathways, particularly the mTORC2 pathway, in controlling tumor growth as well as aspects of tumor malignancy such as invasion and metastasis. 

Here, we provided the first evidence that Rictor is substantially upregulated in a subset of human colon and prostate cancer cell lines. Furthermore, it is upregulated in human colon cancer tissues at a very high incidence (19/20; 95%). To confirm the significance of Rictor upregulation in cancers, we examined human cancer mRNA profiles available at the Gene Expression Omnibus. Rictor is significantly upregulated in endometrial (GSE17025), adrenocortical (GSE12368), and pancreatic carcinoma (GSE15481) relative to non-cancerous tissues. These observations suggest that Rictor upregulation deserves further investigation in a wide variety of human cancers. Together with our previous observation that mTOR kinase is frequently upregulated via activation of oncogenic signaling [32], these findings demonstrate that upregulation of mTORC2 contributes broadly to the progression of human cancers. 

These findings suggest that inhibition of mTOR kinase activity within mTORC2 would be valuable in treatment of cancers. This idea is supported by our observations that downregulation of Rictor suppressed tumorigenicity of cancer cells without any effects on normal cells, whereas downregulation of either Raptor, a component of mTORC1, or mTOR itself strongly suppressed the growth of normal cells (Figure S3). Taken together with the previous finding that deletion of Rictor in a mouse model causes no defects in normal prostate function [16], these observations indicate that selective inhibitors of mTORC2 kinase will be valuable as cancer therapeutics.

In summary, we have demonstrated a critical role for the miR-424/503–Rictor pathway in controlling cancer progression. Considering that this novel axis is activated in both human colon and prostate cancer cells and transformed mouse fibroblasts, it is likely to represent a fundamental mechanism underlying mTOR signaling in cancers. Therefore, study of this pathway should be valuable in the investigation of many types of human cancers. Our findings reveal new avenues for anticancer therapeutic intervention targeted on this pathway.

## Materials and Methods

### Cancer specimens and cell lines

Snap-frozen colon tissues were divided visually into tumor (T) and non-cancerous (N) regions that were then confirmed histologically (see Immunohistochemistry). The research protocol for the collection of human samples was approved by the ethical review board of the Graduate School of Medicine, Osaka University, Japan. Informed consent was obtained from all patients in writing before enrollment in the study. *Csk* ^*-/-*^ mouse embryonic fibroblasts (*Csk* ^*-/-*^ MEFs) were a kind gift from Dr. Akira Imamoto [40]. *Rictor* ^*-/-*^ MEFs and *Rictor* ^*+/+*^ MEFs were kind gifts from Dr. David M Sabatini [11]. Human colon-cancer cell lines (Caco-2, HT-29, HCT116, SW480, and SW620), human prostate-cancer cell lines (PC3, LNCaP, and DU145), normal human prostate cells (PNT1A and PNT2), FHC (normal human colon cells), and HaCaT (normal human keratinocyte cells) were obtained from the American Type Culture Collection (ATCC). MEFs, PC3, and colon cancer cells were cultured in Dulbecco’s modified Eagle’s medium (DMEM). LNCaP, DU145, PNT1A, and PNT2 cells were cultured in RPMI medium. Media were supplemented with 10% fetal bovine serum (FBS). FHC cells were cultured in DMEM/Ham’s F-12 (1:1) with 10% FBS, 5 μg/ml insulin, 5 μg/ml transferrin, and 100 ng/ml hydrocortisone.

### Soft-agar colony-formation assay

Soft-agar colony-formation assay was performed as described [32]. Briefly, single-cell suspensions of 2 × 10^4^ cells were plated in 6-well culture dishes in 1.5 ml of DMEM containing 10% FCS and 0.36% agar on a layer of 2.5 ml of the same medium containing 0.7% agar. Colonies were stained with 3-(4,5-dimethylthiazol-2-yl)- 2,5-diphenyltetrazolium bromide (MTT; Sigma, St Louis, MO, USA) 7-14 days after plating, and micrographs of the stained colonies were used to count the numbers of colonies.

### Tumorigenesis assays

Immunodeficient mice (BALB/c AJc1-nu/nu, CLEA Japan, Inc.) were injected subcutaneously (s.c.) as described [32]. Briefly, mice were injected subcutaneously (s.c.) with 1 × 10^6^ cells suspended in 200 μl of serum-free DMEM at one location. Tumors were monitored every 2 or 3 days and the tumor volume was estimated using the following formula: 0.5 × L × W^2^. At least three mice were used in each experiment. The mice used for this study were housed in environmentally-controlled rooms of the animal experimentation facility at Osaka University and sacrificed under deep anesthesia with isoflurane. All experiments were conducted under the applicable laws and guidelines for the care and use of laboratory animals in the Research Institute for Microbial Diseases, Osaka University, approved by the Animal Experiment Committee of the Research Institute for Microbial Disease, Osaka University.

### Invasion assay

Invasion assays were performed as described [38]. Briefly, Invasion assays were conducted using a BioCoat Matrigel Invasion Chamber (BD Biosciences) according to the manufacturer’s instructions. A cell suspension (1 × 10^5^ cells) in serum-free medium was added to the inserts and each insert was placed in the lower chamber, which contained NIH3T3 cell-conditioned medium. After 48 h of incubation, invasiveness was evaluated by staining the cells that migrated through the extracellular matrix layer. Numbers of invading cells were counted for five microscopic fields per well at a magnification of 100 ×, and the extent of invasion was expressed as the average number of cells per mm^2^.

### Pre-miRNA and anti-miRNA transfection

miR-503 precursor (PM10378), miR-424 (PM10306), antisense miR-503 (AM10378), and anti-miR-424 (AM10306) were purchased from Applied Biosystems. miRNA transfection was performed as described previously [32]. The day before transfection, 2.5 × 10^5^ cells were seeded onto 6-well plates. Different concentrations (5, 15 or 30 nM) of precursor and 30 nM of inhibitor, as well as the negative control, were transfected using Lipofectamine RNAiMAX in 16 μl per 6-well plate according to the manufacturer’s instructions (Invitrogen, Carlsbad, CA, USA). Using this approach, 90% of cells were transfected as judged by comparison to FAM-labeled controls (AM17121; Applied Biosystems).

### Immunochemical analysis

Cells were lysed in n-octyl-β-D-glucoside (ODG) buffer (20 mM Tris-HCl, pH 7.4, 150 mM NaCl, 1 mM EDTA, 1 mM sodium orthovanadate, 20 mM NaF, 1% Nonidet P-40, 5% glycerol, 2% ODG and protease inhibitor cocktail), and immunoblotting was performed as described previously [29]. Immunocytochemistry was performed as described previously [41]. Immunoprecipitation of mTOR complex was performed was carried out as described previously [42]. The following antibodies were used: anti-Src (Ab-1, Calbiochem), anti-Src pY418 (Biosource), anti-Csk, anti-GAPDH (Santa Cruz), anti-Rictor, anti-Raptor, anti-mTOR, anti-AKT pS473, anti-AKT, anti-S6K pT389, anti-S6K (Cell Signaling), and Alexa Fluor 594-conjugated goat anti-mouse IgG (Molecular Probes). The chemical used was Alexa Fluor 488-phalloidin (Molecular Probes).

### Immunohistochemistry

Histologic specimens were fixed in 10% formalin and routinely processed for paraffin embedding. Histological sections 4-μm thick were stained with hematoxylin and eosin and reviewed by two pathologists (JI and EM) to define the cancerous and corresponding normal tissues. An immunoperoxidase procedure was performed on the paraffin-embedded sections as described previously [32]. After antigen retrieval using a Pascal pressurized heating chamber (Dako A/S, Glostrup, Denmark), the sections were incubated with anti-Rictor antibody that was diluted at 1:50. Cells were then treated with a ChemMate EnVision kit (Dako). Diaminobenzidine (Dako) was used as a chromogen. As a negative control, staining was carried out in the absence of primary antibody. Stained sections were evaluated independently by two pathologists (JI and EM).

### Luciferase assay

Luciferase assay was performed as described previously [32]. Two sets of complementary oligonucleotides from the human Rictor 3’UTR containing the putative miR-503 or miR-424 binding sites were designed. The primers used were as follows:

Rictor 3’UTR-1-F:

5’- AGCTTAGCAGATAAGGGCTGTAAGCTGCTGCTTATGTTGAAAAGTGGTTCTTA -3’;

Rictor 3’UTR-1-R:

5’- CTAGTAAGAACCACTTTTCAACATAAGCAGCAGCTTACAGCCCTTATCTGCTA -3’;

Rictor 3’UTR-2-F:

5’- AGCTTTTCTTTTTTATTTAGTAATACGCTGCTACATATTTGGAGGTTCTGGTGA -3’;

Rictor 3’UTR-2-R:

5’- CTAGTCACCAGAACCTCCAAATATGTAGCAGCGTATTACTAAATAAAAAAGAAA -3’;

Rictor-mt 3’UTR-1-F:

5’- AGCTTAGCAGATAAGGGCTGTAAGCGCGGCGGTATGTTGAAAAGTGGTTCTTA-3’;

Rictor-mt 3’UTR-1-R:

5’- CTAGTAAGAACCACTTTTCAACATACCGCCGCGCTTACAGCCCTTATCTGCTA-3’;

Rictor-mt 3’UTR-2-F:

5’- AGCTTTTCTTTTTTATTTAGTAATAGCGGCGGCATATTTGGAGGTTCTGGTGA-3’; and

Rictor-mt 3’UTR-2-R:

5’- CTAGTCACCAGAACCTCCAAATATGCCGCCGCTATTACTAAATAAAAAAGAAA-3’.

### Retroviral gene transfer

Retroviral gene transfer was performed as described previously [27]. Retroviral vectors carrying wild-type chicken c-Src and v-Src were kindly provided by Dr. Tsuyoshi Akagi (KAN Institute, Kobe). 

### Knockdown of genes by shRNA

Lentiviral vectors, empty and carrying human mTOR (ID: NM_004958.2-8344s1c1) were purchased from Sigma. Lentiviral vectors carrying human Rictor, mouse Rictor, and human Raptor were kind gifts from Dr. David M Sabatini. The production of lentiviruses and the infection of cells were performed according to the manufacturer’s instructions.

### Real-time PCR analysis of miRNAs

Expression of mature miRNA was assayed using TaqMan MicroRNA assays (Applied Biosystems) as described previously [32]. miRNA expression was calculated relative to the expression of snoRNA202 (ID: 001232, for mouse) or RNU48 (ID: 001006, for human) (Applied Biosystems). MicroRNA-specific primers for miR-503 (ID: 001048) and miR-424 (ID: 000604) were obtained from Applied Biosystems. 

### Real-time PCR analysis of target gene

Total RNA was prepared using Sepasol (Nacalai Tesque) and reverse transcribed using SuperScript II reverse transcriptase (Invitrogen). The expression of housekeeping gene gapdh was used to normalize the amount of total RNA. Real-time quantitative PCR analysis was performed as described [41]. Specific primers for mouse RICTOR (ID: Mm01307318_m1) and GAPDH (ID: Mm99999915_g1) were obtained from Applied Biosystems. Experiments were carried out in triplicate for each data point.

## Supporting Information

Figure S1 **Role of Rictor on tumor growth of c-Src–transformed cells.** (A) Whole-cell lysates from MEFs were stimulated with or without 10 ng/ml EGF for 4 days and immunoblotted with the indicated antibodies. (B) Whole-cell lysates from Csk^-/-^/c-Src cells expressing control (sh-cont) or Rictor shRNA (sh-Rictor#1, #2, and #3) were immunoblotted with the indicated antibodies. The relative expression levels of Rictor are shown. (C) Soft-agar colony-formation assay for the cells indicated in (B). The mean number of colonies ± S.D. was obtained from three independent experiments. *, p < 0.05 by Student’s t-test. (D) MEFs (Rictor^+/+^) and Rictor-deficient MEFs (Rictor^-/-^) were infected with retrovirus expressing empty vector (Mock) or v-Src. Cell lysates were immunoblotted with the indicated antibodies. (E) Cell lysates from Csk^-/-^ cells (mock) and Csk^-/-^/c-Src cells expressing control (sh-cont) or Rictor shRNA #1 (sh-Rictor) were subjected to immunoprecipitation (IP) with anti-Rictor or anti-mTOR, followed by immunoblotting with the indicated antibodies. The relative expression levels of mTOR and Rictor are shown at the bottom of the panels.
(TIF)

Figure S2 **miR-424/503 cluster as a tumor suppressor of tumor growth.** (**A**) LNCaP cells were treated with 30 nM of miR-503, miR-424, or cont-miR and subjected to the soft-agar colony-formation assay for 8 days. (**B**) Colony-forming activity of LNCaP cells expressing control (sh-cont) or Rictor shRNA. Colonies were scored 8 days after plating. The mean number of colonies ± S.D. was obtained from three independent experiments. *, p < 0.05 and **, p < 0.01 by Student’s t test.
(TIF)

Figure S3 **Rictor downregulation does not affect cell proliferation of human normal epithelial cells.** (A) Whole-cell lysates from HaCaT cells expressing control, mTOR, Raptor or Rictor shRNA (sh-cont, sh-mTOR, sh-Raptor and sh-Rictor, respectively) were immunoblotted with the indicated antibodies. (B) Cell proliferation of the indicated HaCaT cells indicated in (A) was examined by an *in* *vitro* proliferation assay using WST-1. Mean values ± S.D. were obtained from three independent experiments.
(TIF)
