# Supplementary figures and images for: MiR-424/503-Mediated Rictor Upregulation Promotes Tumor Progression
Source: PLoS One. 2013 Nov 11;8(11):e80300. doi: 10.1371/journal.pone.0080300 (PMC3823661; doi:10.1371/journal.pone.0080300)

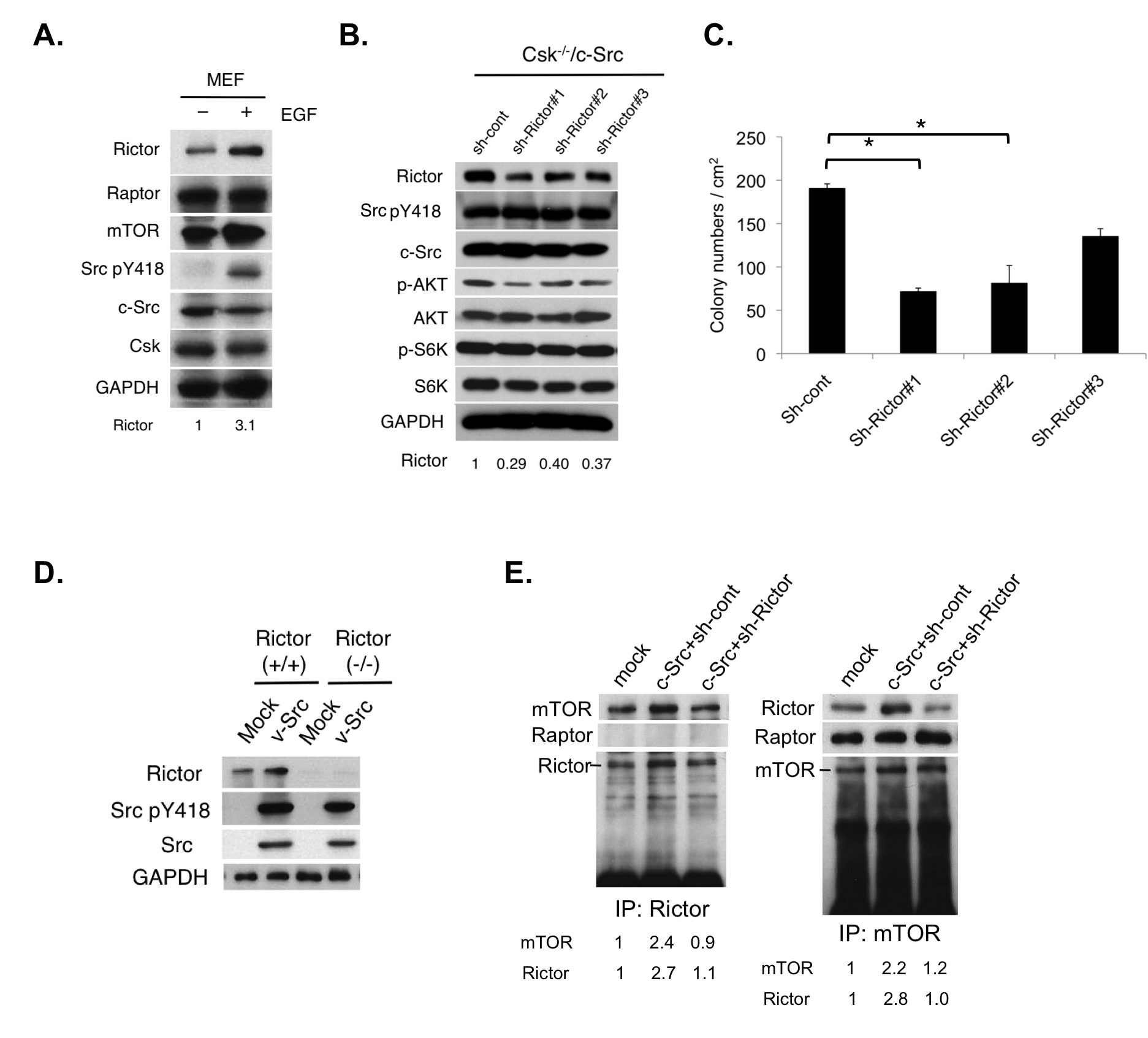

Supplement: Figure S1 — Role of Rictor on tumor growth of c-Src–transformed cells. (A) Whole-cell lysates from MEFs were stimulated with or without 10 ng/ml EGF for 4 days and immunoblotted with the indicated antibodies. (B) Whole-cell lysates from Csk-/-/c-Src cells expressing control (sh-cont) or Rictor shRNA (sh-Rictor#1, #2, and #3) were immunoblotted with the indicated antibodies. The relative expression levels of Rictor are shown. (C) Soft-agar colony-formation assay for the cells indicated in (B). The mean number of colonies ± S.D. was obtained from three independent experiments. *, p < 0.05 by Student’s t-test. (D) MEFs (Rictor+/+) and Rictor-deficient MEFs (Rictor-/-) were infected with retrovirus expressing empty vector (Mock) or v-Src. Cell lysates were immunoblotted with the indicated antibodies. (E) Cell lysates from Csk-/- cells (mock) and Csk-/-/c-Src cells expressing control (sh-cont) or Rictor shRNA #1 (sh-Rictor) were subjected to immunoprecipitation (IP) with anti-Rictor or anti-mTOR, followed by immunoblotting with the indicated antibodies. The relative expression levels of mTOR and Rictor are shown at the bottom of the panels. (TIF) [file pone.0080300.s001.tif]

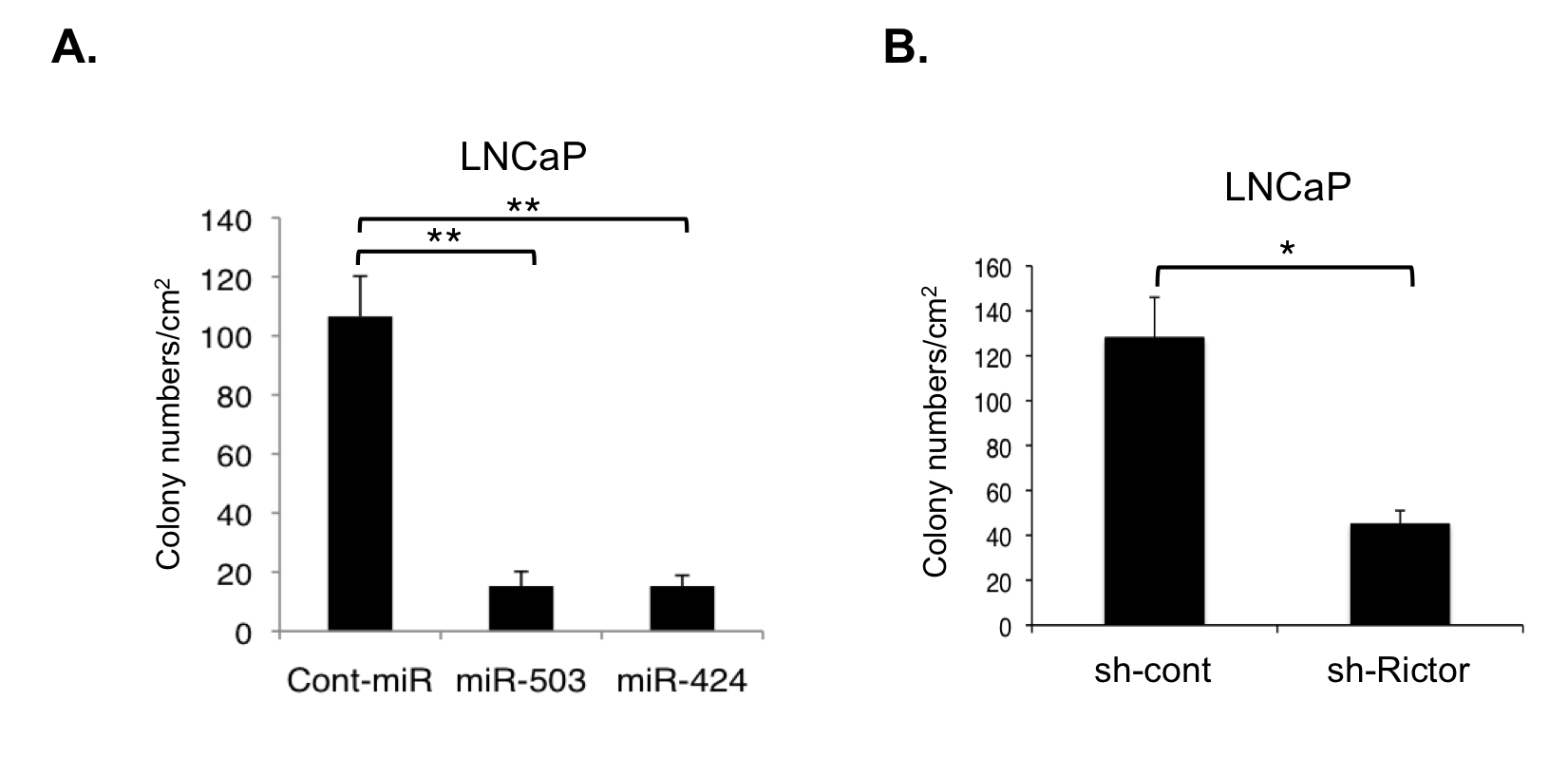

Supplement: Figure S2 — miR-424/503 cluster as a tumor suppressor of tumor growth. (A) LNCaP cells were treated with 30 nM of miR-503, miR-424, or cont-miR and subjected to the soft-agar colony-formation assay for 8 days. (B) Colony-forming activity of LNCaP cells expressing control (sh-cont) or Rictor shRNA. Colonies were scored 8 days after plating. The mean number of colonies ± S.D. was obtained from three independent experiments. *, p < 0.05 and **, p < 0.01 by Student’s t test. (TIF) [file pone.0080300.s002.tif]

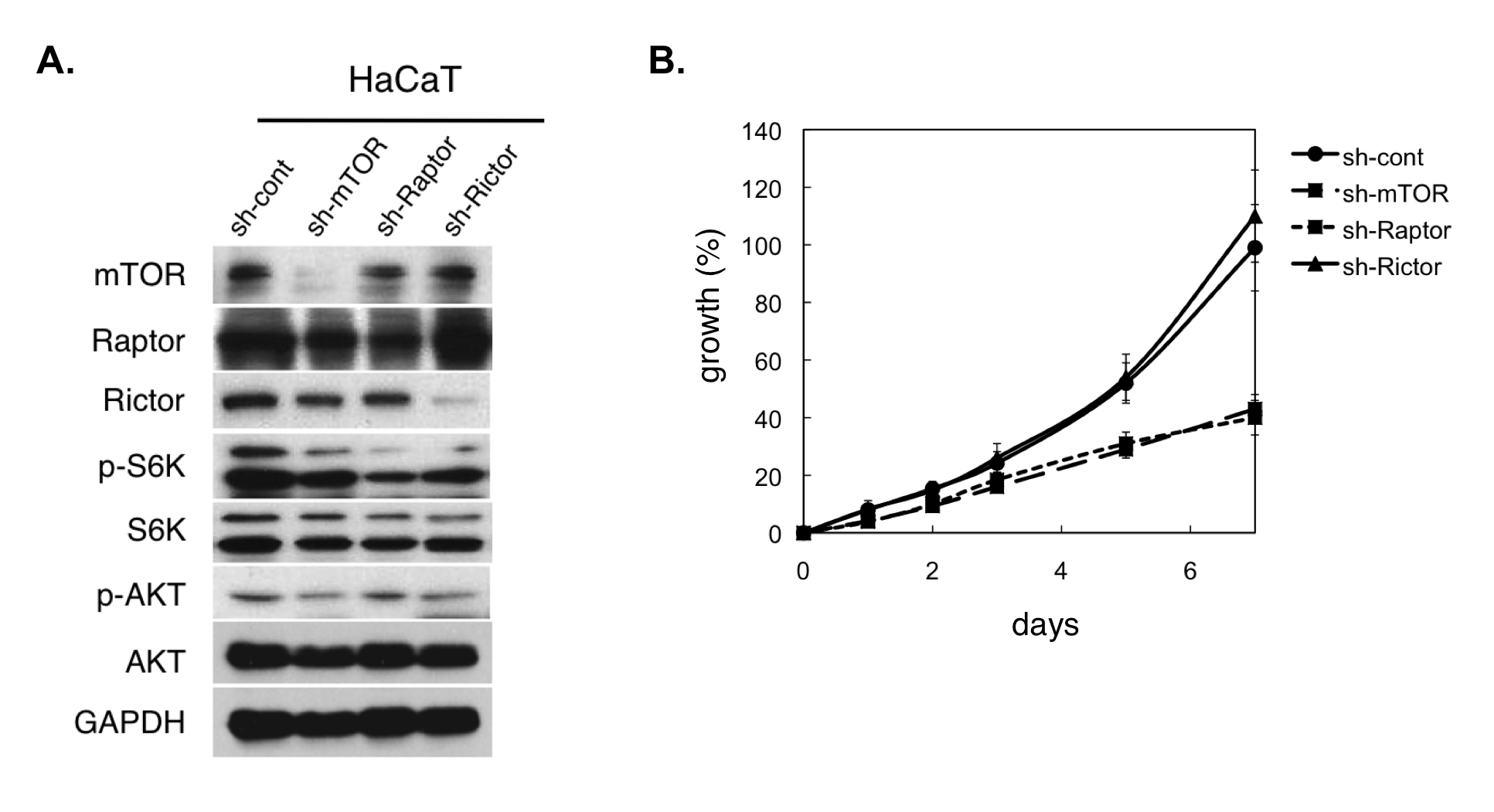

Supplement: Figure S3 — Rictor downregulation does not affect cell proliferation of human normal epithelial cells. (A) Whole-cell lysates from HaCaT cells expressing control, mTOR, Raptor or Rictor shRNA (sh-cont, sh-mTOR, sh-Raptor and sh-Rictor, respectively) were immunoblotted with the indicated antibodies. (B) Cell proliferation of the indicated HaCaT cells indicated in (A) was examined by an in vitro proliferation assay using WST-1. Mean values ± S.D. were obtained from three independent experiments. (TIF) [file pone.0080300.s003.tif]
